# A case of seminal vesicle metastasis from bilateral seminoma

**DOI:** 10.1002/iju5.12813

**Published:** 2024-12-22

**Authors:** Yusuke Aida, Yosuke Yasuda, Emiko Sugawara, Kosuke Takemura, Yusuke Yoneoka, Ryo Fujiwara, Tomohiko Oguchi, Noboru Numao, Junji Yonese, Takeshi Yuasa

**Affiliations:** ^1^ Department of Genitourinary Oncology, Cancer Institute Hospital Japanese Foundation for Cancer Research Ariake, Tokyo Kumamoto Japan; ^2^ Department of Pathology, Cancer Institute Hospital Japanese Foundation for Cancer Research Ariake, Tokyo Kumamoto Japan

**Keywords:** late recurrence, seminal vesicle metastasis, seminoma

## Abstract

**Introduction:**

Postoperative recurrence of seminoma is often seen in lungs, liver, and retroperitoneal lymph nodes, such as the para‐aortic lymph nodes. Most of these recurrences (>90%) occur in the first 3 years after orchiectomy. We report a case of a single recurrence in the seminal vesicle after bilateral radical orchiectomies for metachronous bilateral testicular seminoma.

**Case presentation:**

A 52‐year‐old patient with a past history of bilateral testicular seminoma was diagnosed with left seminal vesicle metastasis by magnetic resonance imaging followed by needle biopsy 8 years after the first orchiectomy. After four cycles of bleomycin, etoposide, and cisplatin regimen were administered, FDG‐18 positron emission tomography‐computed tomography showed no abnormal signal in the left seminal vesicle.

**Conclusion:**

To the best of our knowledge, this is the first report of seminal vesicle metastasis following radical orchiectomies for bilateral testicular seminoma.

**Keynote message**

To the best of our knowledge, this case is the first report of isolated seminal vesicle metastasis following radical orchiectomies for bilateral testicular seminoma.

**Abbreviations & Acronyms**
AFP: alpha fetoprotein
CT: computed tomography
HCG: human chorionic gonadotropin
IGCCCG: International Germ Cell Cancer Collaborative Group
LDH: lactate dehydrogenase
MRI: magnetic resonance imaging
NSGCT: non‐seminomatous germ cell tumor
PET: positron emission tomography
PSA: prostate‐specific antigen
RM: resection margin
18‐FDG: fludeoxyglucose‐18

## Introduction

Seminoma can metastasize either hematogenously or lymphatically. The most common sites of metastases are parenchymal organs, such as the lungs, liver, and retroperitoneal lymph nodes, such as the para‐aortic lymph nodes. Most metastases are seen within 3 years after orchiectomy.^1^ Metachronous contralateral testicular tumors are rare (2.5%).^2^ We report here a case of an isolated recurrence in the seminal vesicle after bilateral radical orchiectomies for metachronous bilateral testicular seminoma.

## Case presentation

**Fig. 1:**
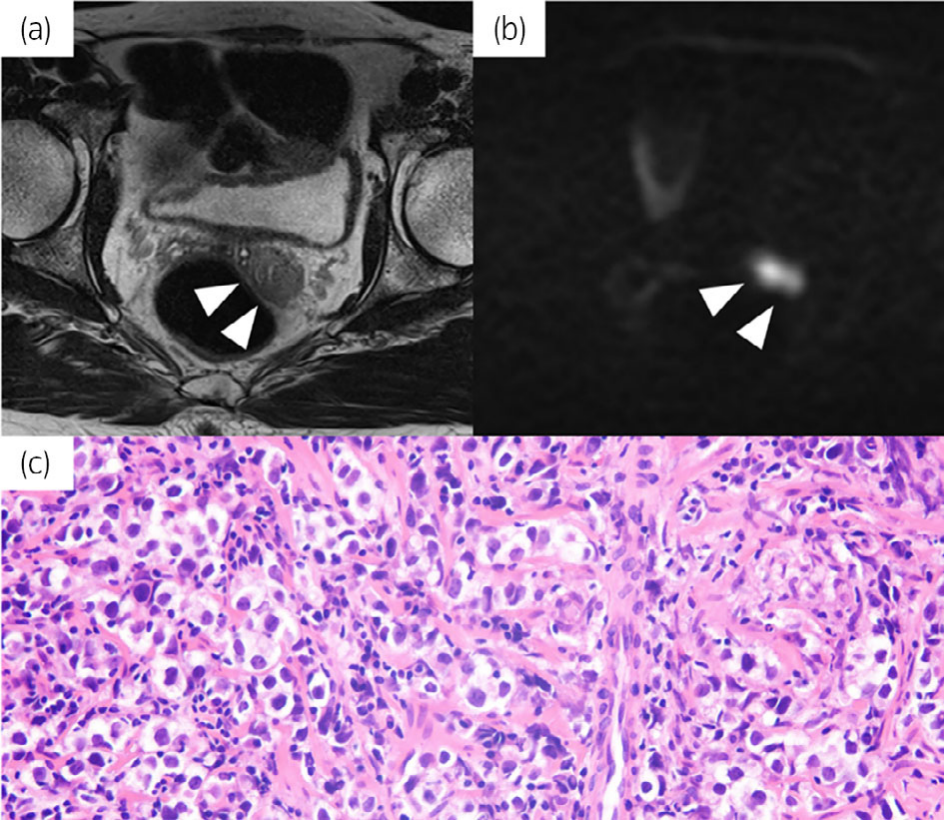
Solitary left seminal vesicle metastasis from testicular seminoma. MRI T2‐weighted (a) and diffusion‐weighted (b) images and histopathological appearance (400× magnification) (c).

We report a case of a 52‐year‐old man who underwent right and left radical orchiectomies at 44 and 48 years of age, respectively. Both pathological examinations disclosed stage 1 seminoma, pT1, RM0 (stage: pT1N0M0S0). After bilateral orchiectomies, he received testosterone replacement therapy. A regular imaging study of thoraco‐abdominal CT scan at 4 years postoperatively showed no metastatic sites, including seminal vesicles, and tumor markers, including HCG, AFP and LDH, were within the normal range. However, the patient complained of urinary discomfort; urine analysis was normal. Digital rectal examination did not show any abnormal induration, and his PSA level was 0.51 ng/mL. MRI revealed no abnormal signal in his prostate. Instead, unexpectedly, T2‐weighted and diffusion‐weighted images demonstrated an abnormal signal in the left seminal vesicle (Fig. 1a,b). Although 18‐FDG PET‐CT was not performed, a trans‐perineum needle biopsy for the seminal vesicle was performed, and all specimens demonstrated seminoma on pathological investigations (Fig. 1c). This case represented solitary left seminal vesicle metastasis 4 and 8 years after left and right radical orchiectomies for testicular seminoma, respectively. Given the presence of non‐pulmonary visceral metastasis, the case was categorized into intermediate prognosis group according to the IGCCCG classification. Subsequently, 4 cycles of bleomycin, etoposide, and cisplatin (BEP) regimen were administered. After chemotherapy, his urinary discomfort improved. In addition, 18‐FDG PET‐CT revealed no abnormal signal, and no new metastatic sites were observed at 4 months after diagnosis of metastasis in the seminal vesicle.

## Discussion

We report a case of solitary seminal vesicle seminoma 4 years after left radical orchiectomy due to testicular seminoma and 8 years after right radical orchiectomy due to testicular seminoma. This case suggests two possible hypotheses in its development, the first being a primary seminal vesicle seminoma. Extragonadal seminoma can develop in many locations, including the intracranial, mediastinum, retroperitoneal, and sacrococcygeal regions. To date, only three cases of primary seminal vesicle seminoma have been reported.^3^, ^4^, ^5^ All of these cases lacked any obvious tumor in the testes and were treated with surgical resection. The second possibility was a seminal metastasis from testicular seminoma. We concluded that our case represented seminal metastasis following bilateral testicular seminoma. One reason for supporting this theory is the extreme rarity of a third primary germ cell cancer. In addition, because this patient had a history of bilateral testicular seminoma, he had possible invisible micro‐metastases outside the seminal vesicle. Ultimately, there are differences in IGCCCG classification between primary seminal vesicle seminoma and metastatic seminoma in the seminal vesicle: namely, any primary extragonadal lesions are classified in a good prognosis group, whereas non‐pulmonary visceral metastases, including the seminal vesicle can be classified in an intermediate prognosis group. To prevent further recurrence, we considered this patient to have an intermediate risk of testicular seminoma, even though this approach could have incurred excessive treatment.

Bilateral testicular germ cell cancers are rare, with a reported incidence between 1% and 5%.^6^, ^7^, ^8^, ^9^ Seminoma is the most frequently (48%) histological type, followed by NSGCT (15%) and malignant lymphoma (22%).^4^ Bilateral testicular cancer is rare, whereas a previous history of testicular cancer is the greatest risk factor, with the contralateral testicle having a 25‐fold increased risk of malignancy.^7^ Thus, it is important to remain vigilant to the possibility of recurrence, not only in the retroperitoneal lymph nodes and lungs, which are the most common sites of metastases, but also in the contralateral testis.

We considered this case to represent left seminal vesicle metastasis 4 years after left testicular seminoma and 8 years after right testicular seminoma, which indicated late or very late relapse of germ cell cancer. Previously, we reported the recurrence rate of testicular germ cell cancer with surveillance policy.^10^ In the 5‐year observation period of that study, recurrence rates were 9.3% (5/54) in patients with seminoma and 10% (3/30) in patients with NSGCT.^10^ All of these patients experienced a recurrence within 2 years; all underwent induction chemotherapy and remained alive with no evidence of disease.^10^

Although solitary seminal vesicle metastases are extremely rare entities, some case reports have identified metastases from hepatocellular carcinoma, renal cell carcinoma, and ileal adenocarcinoma.^11^, ^12^ These cases seem to represent lymphatic or vascular spread to the seminal vesicle. Our case might represent metastasis via the seminal duct.

Cases of primary seminoma of seminal vesicle have been treated by radical prostatectomy or radical cystoprostatectomy.^3^, ^4^, ^5^ In our case, however, chemotherapy was chosen because of the decreased quality of life associated with surgery. However, treatment of seminoma with seminal vesicle as the primary or metastatic site warrants further study.

In conclusion, we report a case of seminoma that metastasized to the left seminal vesicle. To the best of our knowledge, this is the first report of seminal vesicle metastasis following radical orchiectomies for bilateral testicular seminoma.

## Author contributions

Yusuke Aida: Writing – original draft. Yosuke Yasuda: Supervision; writing – review and editing. Emiko Sugawara: Resources; writing – review and editing. Kosuke Takemura: Supervision; writing – review and editing. Yusuke Yoneoka: Resources; writing – review and editing. Ryo Fujiwara: Supervision; writing – review and editing. Tomohiko Oguchi: Resources; writing – review and editing. Noboru Numao: Supervision; writing – review and editing. Junji Yonese: Supervision; writing – review and editing. Takeshi Yuasa: Supervision; writing – review and editing; project administration.

## Conflict of interest

The authors declare no conflict of interest.

## Approval of the research protocol by an Institutional Reviewer Board

Not applicable.

## Informed consent

Informed consent from the patient was obtained.

## Registry and the Registration No. of the study/trial

Not applicable.
